# Nodular Meningeal Recurrence After Laser Interstitial Thermal Therapy for Brain Metastasis: Diagnostic Challenges and Role of Reirradiation With Preoperative Fractionated Stereotactic Radiation Therapy

**DOI:** 10.1016/j.adro.2026.102046

**Published:** 2026-04-01

**Authors:** Marshall Harrell, Tugce Kutuk, Alexander Crum, Natalie Clark, Joshua Palmer, Raju Raval, Sasha Beyer, Simeng Zhu, John Grecula, Dukagjin Blakaj, James Bradley Elder, Raj Singh

**Affiliations:** a Department of Radiation Oncology, The Ohio State University Wexner Medical Center, Columbus, Ohio; b Department of Neurosurgery, The Ohio State University Wexner Medical Center, Columbus, Ohio; c Department of Radiation Oncology, Lynn Cancer Institute, Baptist Health South Florida, Boca Raton, Florida

## Introduction

Nodular meningeal disease (NMD) is a clinically significant recurrence pattern characterized by discrete meningeal tumor involvement, most commonly arising along the dura, and is strongly associated with neurosurgical resection followed by adjuvant stereotactic radiosurgery (SRS).^1^ Historically, whole-brain radiation therapy (WBRT) was the standard postoperative treatment, providing comprehensive coverage of microscopic disease beyond the resection cavity.^2^ However, concerns over delayed neurocognitive toxicity have driven a shift away from WBRT toward focal approaches such as SRS and fractionated stereotactic radiation therapy (FSRT).^3^ With this transition, the incidence of meningeal patterns of recurrence has increased.^3^ NMD is distinct from diffuse leptomeningeal disease (LMD), which represents a cerebrospinal fluid-dominant process with linear leptomeningeal enhancement; in this report, NMD refers to a localized, dural-based nodular recurrence.

Laser interstitial thermal therapy (LITT) is increasingly used as a minimally invasive option for enlarging and often symptomatic intracranial lesions following prior radiation, providing therapeutic cytoreduction to decrease local lesion burden and associated edema, while enabling stereotactic biopsy for tissue diagnosis when the distinction between radiation necrosis (RN) and tumor recurrence remains uncertain.^4^ Although LITT carries a low but measurable risk of tract seeding, estimated at 5.4%, NMD has not been previously reported following this procedure.^5^ Similar to surgical resection, disruption of the blood-brain barrier and blood-dura barrier during biopsy or ablation may also predispose to nodular meningeal dissemination.^4^

Here, we present what is, to our knowledge, the first reported instance of NMD after LITT in small cell lung carcinoma (SCLC) brain metastasis. This case highlights the diagnostic challenges of distinguishing RN from tumor recurrence, where an initial brain magnetic resonance imaging (MRI) scan with perfusion suggested active disease, while an ¹⁸F-fluciclovine positron emission tomography (PET) scan and biopsy favored RN, only for subsequent progression to confirm viable tumor. Moreover, it raises concern for potential dural seeding after minimally invasive procedures, such as biopsy and LITT, and highlights how preoperative SRS or FSRT can be strategically integrated into salvage management for NMD recurrence.

## Case Presentation

A 62-year-old man with a history of limited-stage SCLC of the right upper lobe, initially treated with definitive chemoradiation therapy (45 Gy in 30 twice-daily fractions), subsequently developed extensive-stage disease with brain metastases. He underwent Gamma Knife (Elekta) SRS, 20 Gy in 1 fraction, to right frontal, left parietal, and right posterior temporo-occipital metastases, all achieving a complete radiographic response. A right parietal metastasis was later treated with linear accelerator-based SRS, 20 Gy in 1 fraction, with an initial radiologic complete response on a 2-month follow-up brain MRI scan. He remained off systemic therapy, with a stable left adrenal metastasis and no other active extracranial disease.

**Figure 1:**
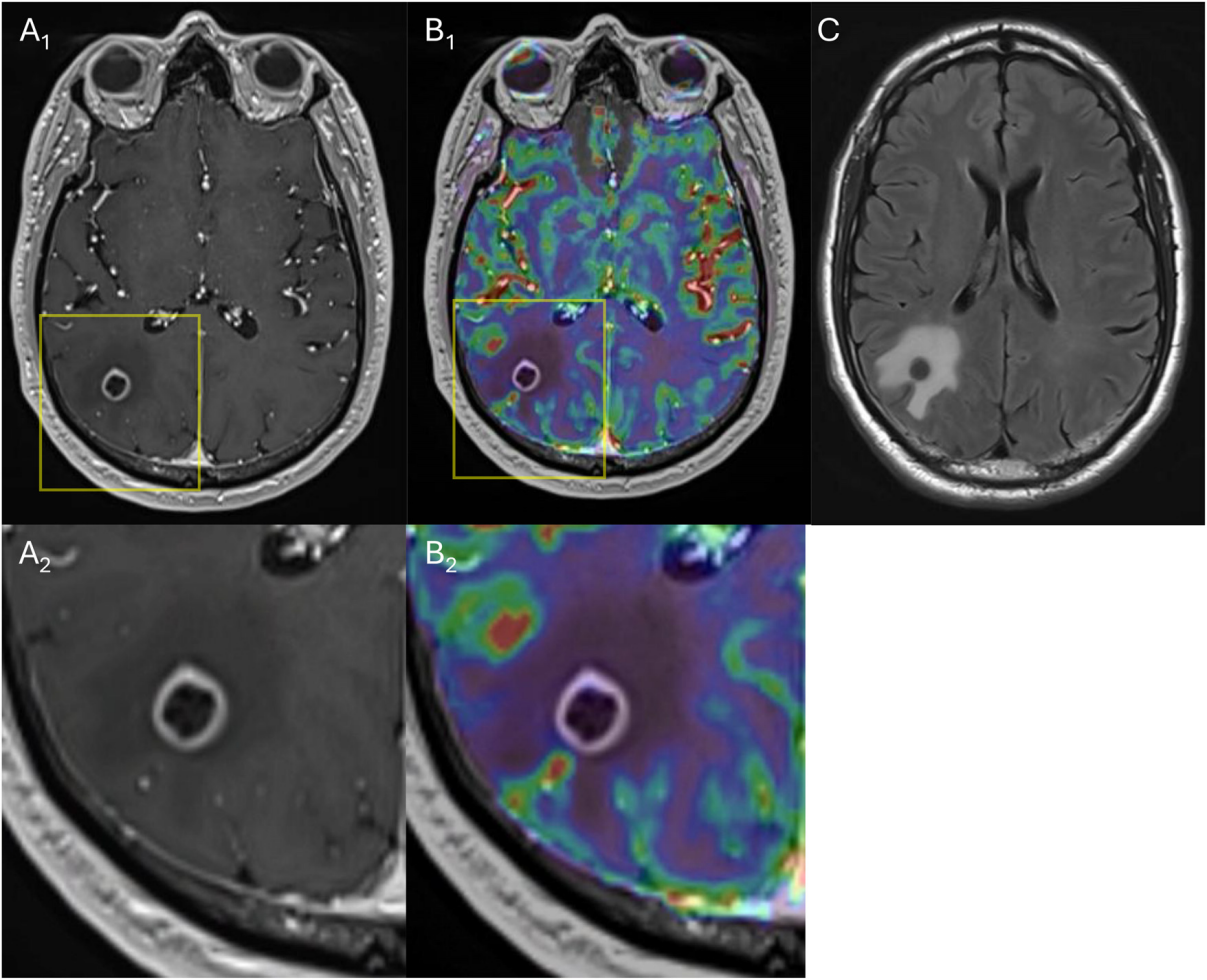
A magnetic resonance imaging (MRI) scan of the brain with dynamic susceptibility contrast (DSC) perfusion performed 8 months after linac-based stereotactic radiosurgery (SRS). (A_1_) Axial T1-weighted postcontrast (T1c+) demonstrates a 1.3 × 1.3 cm peripherally enhancing lesion in the right parietal lobe; the yellow box denotes the magnified view in (A_2_). (B_1_) DSC perfusion overlay demonstrates low perfusion within the enhancing components; the yellow box denotes the magnified view in (B_2_). (C) Axial T2 fluid-attenuated inversion recovery (FLAIR) demonstrates surrounding edema.

**Figure 2:**
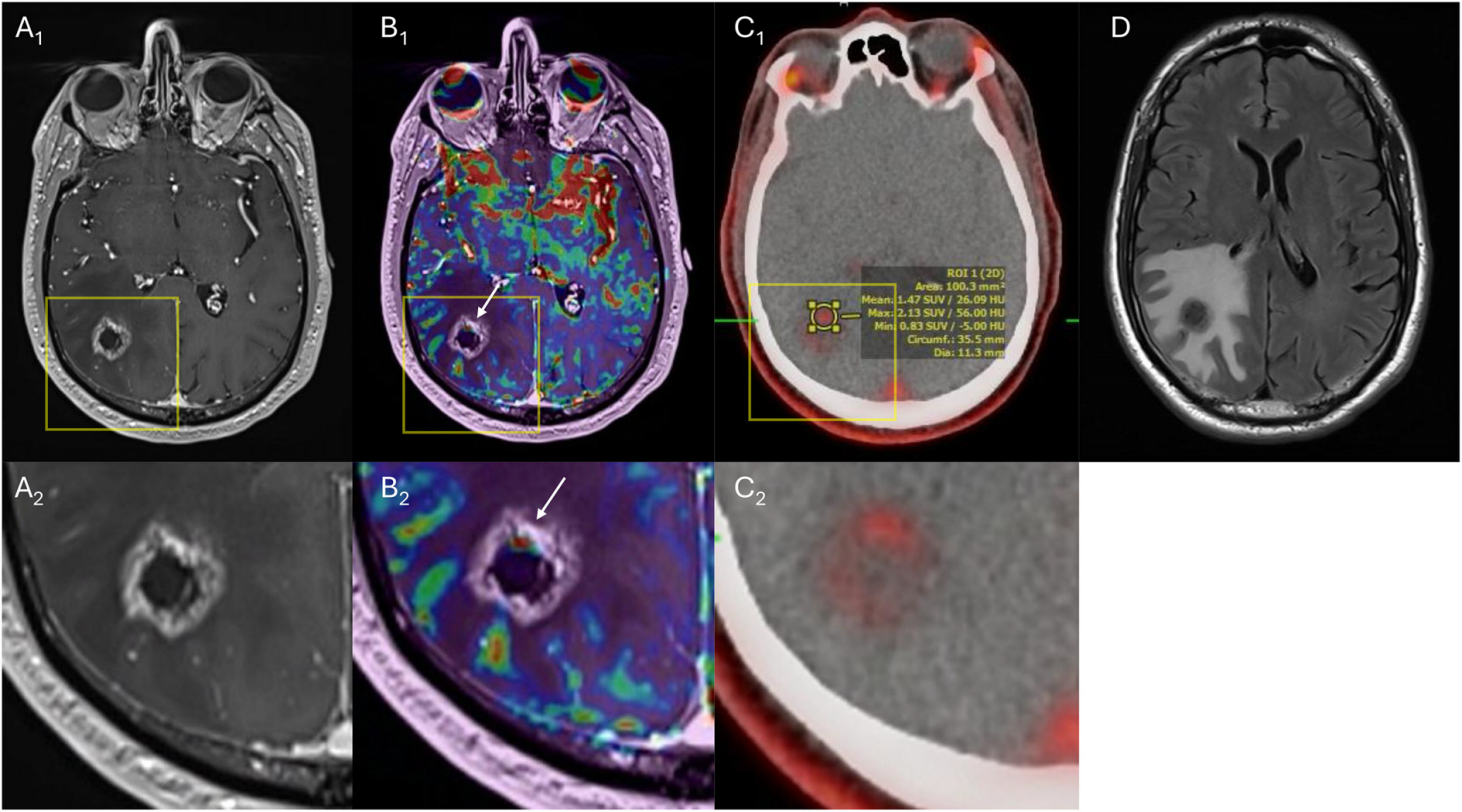
A magnetic resonance imaging (MRI) brain with dynamic susceptibility contrast (DSC) perfusion and an ¹⁸F-fluciclovine positron emission tomography/computed tomography (PET/CT) scan performed approximately 12 months after linac-based stereotactic radiosurgery (SRS). (A_1_) Axial T1c+ demonstrates a 2.3 × 2.1 cm peripherally enhancing lesion; the yellow box denotes the magnified view in (A_2_). (B_1_) DSC perfusion overlay demonstrates increased perfusion within the anterior enhancing components (white arrow); the yellow box denotes the magnified view in (B_2_). (C_1_) An ¹⁸F-fluciclovine PET/CT scan demonstrates low radiotracer uptake (maximum standardized uptake value [SUVmax] 2.13); the yellow box denotes the magnified view in (C_2_). (D) Axial T2 fluid-attenuated inversion recovery (FLAIR) demonstrates increased surrounding edema.

Eight months post-SRS, a surveillance MRI scan with dynamic susceptibility contrast (DSC) perfusion demonstrated mild enlargement of the treated right parietal lesion with low perfusion, favoring RN (Fig. 1). He was started on Boswellia 4800 mg daily, but this was discontinued because of gastrointestinal intolerance. On a repeat MRI scan at 12 months, the lesion demonstrated further enlargement with increased vasogenic edema and elevated relative cerebral blood volume in the enhancing components on DSC perfusion, suggesting a possible active tumor (Fig. 2). However, a ¹⁸F-fluciclovine PET scan revealed low uptake (maximum standardized uptake value [SUVmax] 2.1), supporting RN. Given progressive lesion enlargement with worsening edema and persistent diagnostic uncertainty, MRI scan-guided LITT with stereotactic biopsy was performed, with pathology resulting in necrosis.

**Figure 3:**
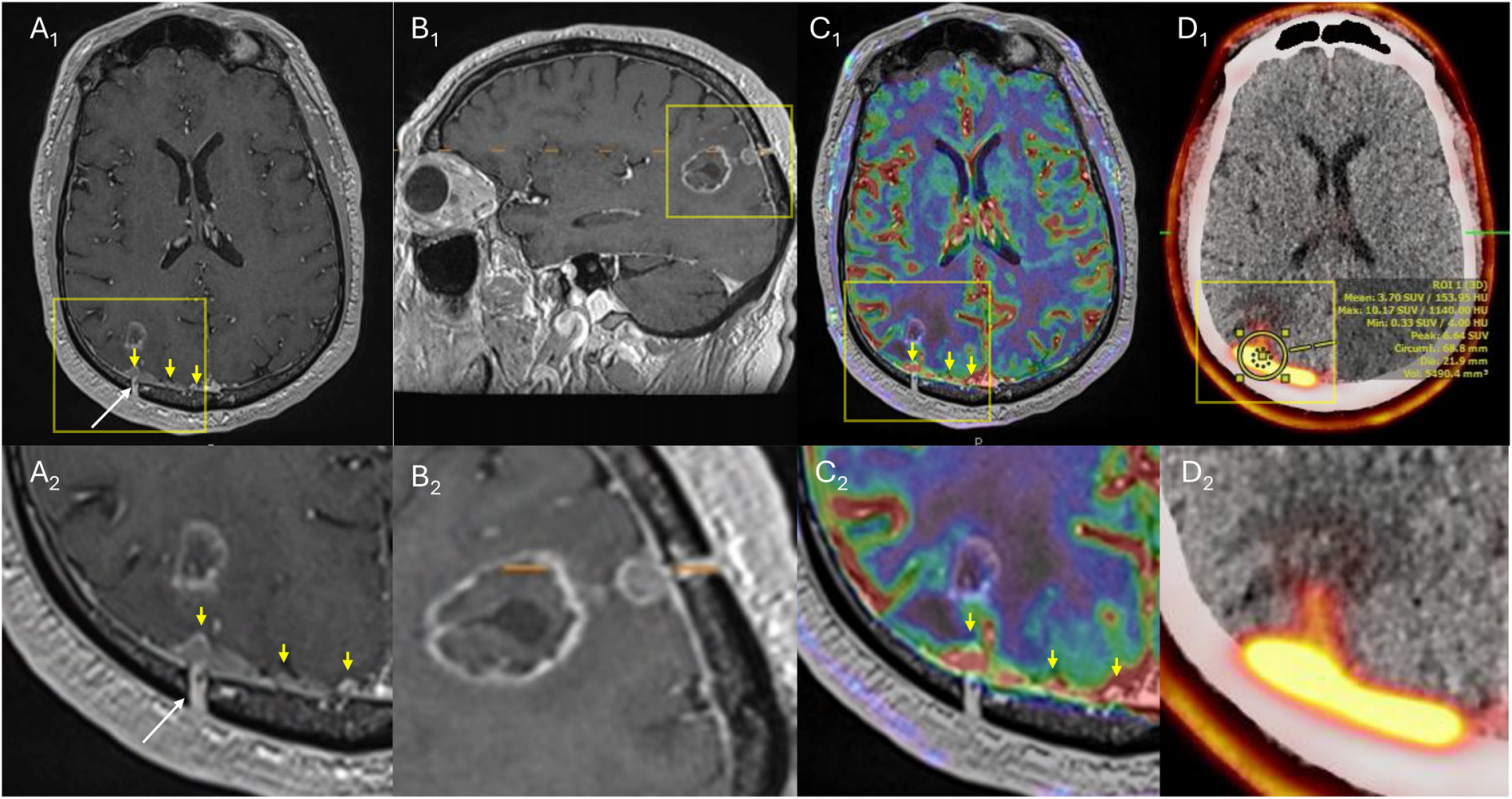
A magnetic resonance imaging (MRI) brain with dynamic susceptibility contrast (DSC) and an ¹⁸F-fluciclovine positron emission tomography/computed tomography (PET/CT) scan performed approximately 4 months after laser interstitial thermal therapy (LITT) (17 months after linac-based stereotactic radiosurgery [SRS]). (A_1_) Axial T1c+ demonstrates post-surgical changes along the right parietal burr-hole craniotomy (white arrow) with nodular dural enhancement immediately subjacent to the craniotomy (yellow arrows); the yellow box denotes the magnified view in (A_2_). The superior aspect of the resection cavity, seen more anteriorly, remains stable. (B_1_) Sagittal view demonstrates the stable resection cavity, dural nodularity, and burr-hole–related surgical changes; the orange dotted line denotes the axial level corresponding to the T1c+ and perfusion images. (C_1_) DSC perfusion overlay demonstrates increased perfusion corresponding to areas of nodular dural enhancement (yellow arrows); the yellow box denotes the magnified view in (C_2_). (D_1_) An ¹⁸F-fluciclovine PET/CT scan demonstrates intense radiotracer uptake (maximum standardized uptake value [SUVmax] 10.17); the yellow box denotes the magnified view in (D_2_).

Within 2 months of LITT, the patient developed new-onset seizures, accompanied by mild headaches and subtle cognitive changes. An MRI scan of the brain revealed several new dural-based enhancing lesions with increased perfusion along the posterior parieto-occipital region with possible intraparenchymal involvement adjacent to the previously treated parietal lesion (Fig. 3). These findings were most consistent with NMD. A ¹⁸F-fluciclovine PET scan demonstrated intense uptake (SUVmax value 10.1) in the right parietal dura, corresponding to the MRI abnormality, strongly favoring active metastatic disease.

**Figure 4:**
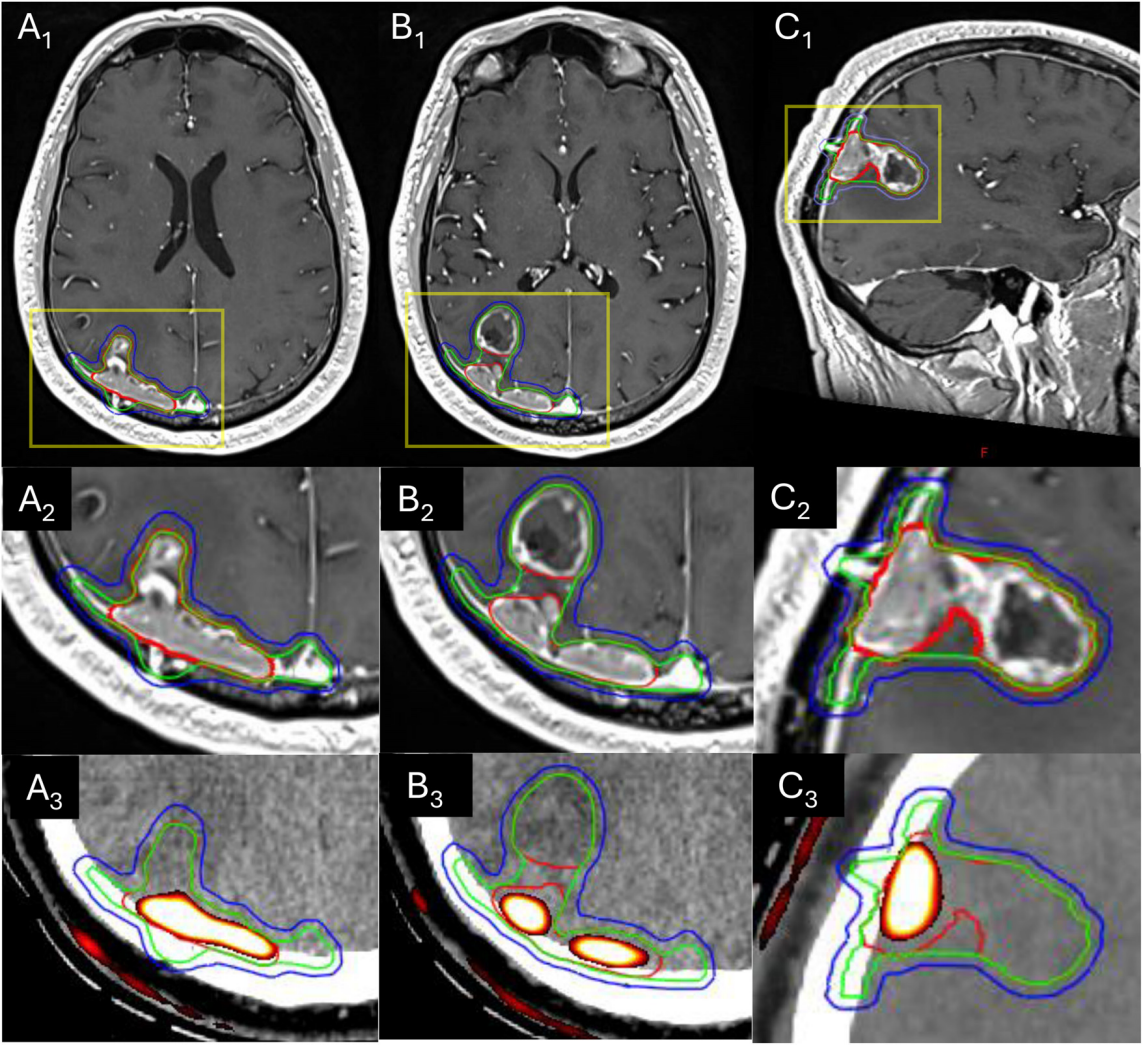
Preoperative reirradiation target volumes for the right parietal lesion with a magnetic resonance imaging (MRI) scan T1c+ and an ¹⁸F-fluciclovine positron emission tomography (PET) scan fusion. Gross tumor volume (GTV, red) encompasses the prior laser interstitial thermal therapy (LITT) tract and operative changes, contrast-enhancing regions, nodular dural disease on an MRI scan, and hypermetabolic regions on an ¹⁸F-fluciclovine PET scan. Clinical target volume (CTV, green) represents an anatomically tailored 3 to 5 mm expansion along the meninges with inclusion of the superior sagittal sinus. Planning target volume (PTV, blue) represents a 2 mm expansion from the CTV. Yellow boxes in A_1_, B_1_, and C_1_ denote magnified views in the middle and lower rows. (A_1_) Full axial MRI scan T1c+ fusion at the level of the burr hole and LITT tract, with a corresponding magnified MRI scan T1c+ view in (A_2_) and planning computed tomography (CT)/PET scan overlay in (A_3_). (B_1_) Full axial MRI scan T1c+ fusion at a more inferior level of the lesion, where the cavitary parenchymal component is more distinct from the nodular dural disease; corresponding magnified views of T1c+ and DSC perfusion overlays are shown in (B_2_) and (B_3_), respectively. (C_1_) Sagittal MRI scan T1c+ at the level of the burr hole and LITT tract, with enhancing parenchymal and nodular dural disease encompassed within the target volumes; corresponding magnified views of T1c+ and DSC perfusion overlays are shown in (C_2_) and (C_3_), respectively.

**Figure 5:**
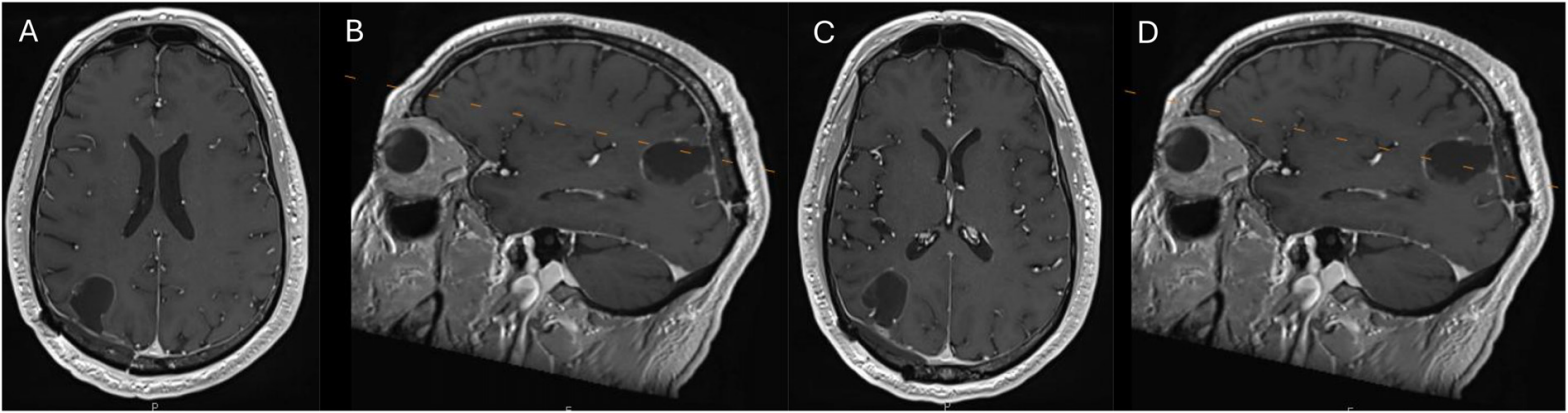
A magnetic resonance imaging (MRI) scan of the brain was performed 2 months after preoperative reirradiation and surgical resection. Postoperative changes are present in the right parietal lobe with improvement in surrounding edema and no evidence of progressive disease at treated sites or new brain metastases. (A) Axial MRI scan at the level of the superior aspect of the lesion. (B) Sagittal view with the orange dotted line denoting the level in (A). (C) Axial T1c+ at the level of the inferior aspect of the lesion. (D) Sagittal view with the orange dotted line denoting the level in (C).

At the multidisciplinary tumor board, the consensus recommendation was to pursue reirradiation with preoperative FSRT followed by resection. He subsequently underwent FSRT, 24 Gy in 3 fractions (Fig. 4), followed by surgical resection, with final pathology revealing metastatic SCLC adherent along the LITT tract and the adjacent dura, with necrosis and astrogliosis in the deeper intra-axial tissue. At the 2-month follow-up, an MRI scan with DSC perfusion demonstrated stable postoperative changes with improved edema, no new metastases, and no evidence of disease progression (Fig. 5).

## Discussion

This case underscored several important clinical lessons. First, it demonstrates the potential risk of NMD following LITT. Although NMD has traditionally been associated with surgical resection via craniotomy followed by postoperative SRS, our findings suggest that even minimally invasive procedures, such as LITT, may also carry similar risks. The intraoperative observation of the tumor adherent to the dura along the LITT tract supports the hypothesis that the disruption of the blood-brain barrier and blood-dura barrier during ablation or biopsy can create a conduit for dural seeding.^4^^,^^5^ This expands the spectrum of procedures associated with meningeal recurrence and emphasizes vigilance when applying minimally invasive techniques for brain metastasis and presumed RN treatment. Additionally, stereotactic biopsy of previously irradiated, heterogeneous lesions may yield false-negative results because of spatial sampling error, whereby focal areas of viable tumor may be missed.

Second, this case highlighted the diagnostic complexity of differentiating RN from tumor recurrence. A conventional brain MRI scan is often limited because both RN and recurrent tumors enhance and generate perilesional edema. Advanced MRI scan perfusion techniques can aid in this distinction. In a meta-analysis, dynamic contrast-enhanced perfusion achieved a pooled sensitivity of 74% and specificity of 92%, whereas DSC perfusion showed a sensitivity of 83% and specificity of 78%.^6^ Despite these advances, institutional experiences continue to demonstrate their limitations, with histopathology often required for confirmation.^7^^,^^8^ Similarly, an ¹⁸F-fluciclovine PET scan has shown promise in distinguishing RN from tumor recurrence^.9^ A prospective pilot study from the Cleveland Clinic reported that higher SUVmax values were predictive of progression, with a mean SUVmax value of 8.59 in tumor progression versus 4.18 in RN and an overall area under the curve value of 0.875. An SUVmax value cutoff of 4.3 provided 100% sensitivity and 63% specificity.^9^ In our case, however, results were initially discordant. Although the initial report of increased perfusion on brain MRI scan was concerning for an active tumor, the ¹⁸F-fluciclovine PET scan demonstrated low uptake (SUVmax value 2.1), favoring RN, which was also supported by pre-LITT biopsy findings. At the time of nodular meningeal recurrence following LITT, a conventional MRI scan’s findings were diagnostic, with DSC perfusion and an ¹⁸F-fluciclovine PET scan providing complementary information that increased diagnostic confidence in the context of prior discordant imaging and biopsy results (SUVmax value 10.1).

Additionally, this case highlighted the rationale for preoperative stereotactic radiation in a salvage setting. Delivering SRS or FSRT preoperatively may sterilize tumor cells before disruption of dural and other tissues along the operative tract, thereby reducing the risk of subsequent meningeal spread, while also decreasing rates of RN through irradiation of smaller intact volumes with subsequent removal of high-risk irradiated tissue at resection.^10^, ^11^, ^12^ Growing evidence from institutional series and prospective data has demonstrated lower rates of meningeal recurrence and RN, with comparable local control and survival, using preoperative compared with postoperative approaches.^10^, ^11^, ^12^ In the present case, preoperative FSRT was selected, given concern for further meningeal dissemination with surgery and postoperative irradiation. Early posttreatment imaging demonstrated no evidence of progression; however, follow-up remains limited, and marginal or distant meningeal recurrence remains possible, underscoring the importance of continued close surveillance.

In cases where a viable tumor is identified on biopsy following LITT without evidence of NMD, post-LITT SRS may be considered; however, data guiding optimal target delineation, including whether to encompass the ablation cavity or LITT tract, remain limited, particularly given the frequent lack of tract visualization on MRI scan. In this setting, management decisions are typically guided by symptom burden, with consideration of preoperative radiation and surgery in symptomatic patients versus short-interval surveillance in those who are asymptomatic.

Most prior series reported meningeal disease collectively, rather than distinguishing LMD and NMD, making the true incidence of NMD less well defined.^13^ In a systematic review and meta-analysis, Lamba et al^13^ reported a nearly 3-fold higher risk of LMD with postoperative SRS compared with WBRT (risk ratio, 2.99; 95% confidence interval, 1.55-5.76), although NMD rates did not significantly differ between modalities.^13^ Shi et al^14^ reported regional recurrence and LMD rates of 15.8% following postoperative SRS. Mahajan et al^15^ described 1-year risks of nodular meningeal recurrence approaching 28%. Importantly, multiple studies have linked meningeal recurrence to poor survival and increased risk of neurologic death.^1^^,^^11^^,^^16^ A recent systematic review and meta-analysis reported the prevalence of meningeal recurrence (combined LMD and NMD) at 20.9%, with NMD accounting for 54.6% of cases.^17^ Reported NMD rates after resection and postoperative SRS range from 8% to 21.5%, with risk factors including piecemeal resection and direct tumor contact with pia or dura.^1^^,^^17^ Prior irradiation is another adverse factor; resection of previously treated lesions carries more than double the risk of NMD compared with radiation-naïve lesions (hazard ratio, 2.3; 95% confidence interval, 1.25-4.57; *P* = .008).^1^ Preoperative radiation is being investigated as a strategy to mitigate these risks by sterilizing tumor cells before resection and reducing RN through treatment of smaller volumes, with subsequent removal of high-risk irradiated tissues.^10^^,^^11^

In summary, this case highlighted the potential risk of NMD following LITT, the diagnostic complexity of differentiating RN from tumor recurrence, and the therapeutic value of preoperative SRS or FSRT in managing previously irradiated recurrent disease. Because utilization of LITT expands for both suspected RN and tumor recurrence, clinicians should remain vigilant for the possibility of NMD, especially in aggressive histologies, such as SCLC. Larger institutional and prospective studies are needed to clarify the incidence of NMD and optimize management strategies after minimally invasive interventions.

## Disclosures

Joshua Palmer receives grants from Genentech and the National Institute of Health (NIH); consulting fees from ICOTEC; honoraria from Varian Medical Systems, Novocure, and ICOTEC; travel stipend from Varian Medical Systems and ICOTEC; advisory board on DxCover; executive committee member for Spine Therapy Society. John Grecula receives grants from IntraOp Medical Corporation. Dukagjin Blakaj receives grants from IntraOp Medical Corporation. Tugce Kutuk received travel stipend from Gammatile. Marshall Harrell received travel stipend from Gammatile. The other authors declare that they have no known competing financial interests or personal relationships that could have appeared to influence the work reported in this paper.
